# Lyrics and artistic improvisations in health promotion for the COVID-19 pandemic control in East Africa

**DOI:** 10.1177/1757975920973671

**Published:** 2020-12-01

**Authors:** Benson A. Mulemi

**Affiliations:** Department of Social Sciences and Development Studies, Catholic University of Eastern Africa, Nairobi, Kenya

**Keywords:** COVID-19, digital lyrics, East Africa, health promotion, Kenya, public, social media

## Abstract

News about the outbreak of coronavirus disease 2019 (COVID-19) in Wuhan City, Hubei Province, China in December 2019 diffused gradually to East Africa through mainstream media and social media. The general public construed the pandemic threat as being ‘far away’ and associated it with foreign practices and behaviours. Social media discourse was initially replete with indifference about the perceived risk. Conflicting views about the possibility of the pandemic spreading to Africa and the complexity of explaining its causes delayed the desired understanding of the reality of the global public health concern. The popular public response to the COVID-19 control discourse is therefore characterised by ambivalence about embracing the pandemic control protocols. Drawing on content and discourse analysis of musical and poetic compositions on COVID-19 by artists in East Africa and shared among WhatsApp users in Kenya, this article describes local perspectives on COVID-19 risk and their health promotion implications. It explores local construction of the meaning of the COVID-19 pandemic and its consequences for effective health promotion. The article considers the spontaneous musical and poetic performances by experienced and amateur artists as local attempts to enhance compliance with the global COVID-19 control protocols and popular participation in local health promotion. The basic premise is that artists’ creation and sharing of digital COVID-19 lyrics denote their attempt to go beyond the medical logic of health promotion by including broad aspects of a cultural logic of care. This approach would establish an integrated and sustainable health promotion framework to prevent the spread of COVID-19 and its impact on local societal wellbeing.

## Introduction

African artists adapted fast to the increasing global anxiety and uncertainty about the coronavirus disease 2019 (COVID-19) pandemic through improvisation and sharing of catchy songs and lyrics. The artistic compositions and performances shared as digital videos aim at promoting awareness about the global pandemic and the urgency of its prevention. The lyrical performances in various local styles have been shared widely through social media channels in East Africa as short digital videos. The initial focus of the lyrics was on creating awareness about personal hygiene, staying at home and keeping social distance as important aspects of the coronavirus pandemic control protocols emphasised since mid-February 2020. The news about the outbreak of COVID-19 in Wuhan City, Hubei Province, China, in December 2019 diffused rather slowly to East Africa through mainstream and social media channels. Government ministries of health relayed the medical interpretation of the news about the disease as a new severe acute respiratory syndrome dubbed coronavirus-2 (SARS-CoV-2). East African governments emphasised the exigent implementation of the COVID-19 prevention and treatment protocols as recommended by the World Health Organization (WHO). However, effective prevention and management of a global pandemic of this nature depends on the extent to which the participation of the wider general public can be mobilised in local contexts. A general lag in official response to the disease and effective conscription of public participation in health promotion for the control of the coronavirus spread defined the relatively low public preparedness to cope with the pandemic in Africa. The masses in East Africa understood COVID-19 risk as being ‘far away’ from the continent. The public associated perceived vulnerability to the pandemic with exotic practices and behaviours which were unlikely in local cultures. Social media activity initially presented indifference to the WHO and national governments’ earnest cautionary messages about an imminent global coronavirus pandemic. Conflicting views about the possible origin of COVID-19, the possibility of its spread to Africa and the complexity of explaining its causes shaped the lag in providing culturally appropriate and effective communication about the reality of the global public health risk. The January 30, 2020 WHO declaration of the outbreak as a Public Health Emergency, with significant implication for international health, evoked a sense of heighted alert in Africa. Formal mass media channels intensified news segments depicting an appalling global rapid increase in COVID-19-related deaths and acute illnesses. The daunting excerpts were rapidly relayed to the public through popular conventional mass media and social media channels with an aim of increasing awareness about the pandemic risk. The ominous news did not seem to elicit a sense of urgency in the general public to readily adopt the WHO COVID-19 control protocols as communicated by national government ministries of health. Initial coronavirus lyrics and artistic improvisations for health promotion in East Africa, therefore, included news excerpts of experiences of the disease in non-African settings.

## Background

Relative complacency about COVID-19 risk and public susceptibility in East Africa lingered in spite of the confirmation of the global surge of cases since February 2020. Local media coverage of disturbing scenarios from the epicentres of the disease in China, Italy, Iran and some states in the USA did not evoke the expected awareness and health behaviour among a large section of the general public. The onset of intense political visibility of the disease in East Africa was in mid-March 2020, when the first case was reported in Kenya and subsequently in the neighbouring countries. Response to the looming public health calamity entailed panic and frantic government efforts to secure the public. However, most local communities did not readily internalise the meaning and implications of the pandemic as presented through popular formal and social media channels accessible to them. Uncertainty and conflicting views about the local magnitude, risk and prognosis of the global COVID-19 pandemic shaped desperate health promotion efforts in East Africa. Popular coronavirus discourse at the onset of the pandemic entailed perceptions about either insignificant or unknown COVID-19 risk and vulnerability of local populations due to various reasons. The alert sent to the WHO on December 31, 2019 by the China Health Authority of cases of pneumonia reported in Wuhan City on December 8, 2019, for instance, indicated that the aetiology of the illness attributed to coronavirus was actually unknown. However, many patients worked at or lived around the local Huanan Seafood Wholesale Market (1,2) which was widely considered to be the origin of the virus. Conversely, not all the earlier pneumonia cases had a connection to the seafood market, yet the common narrative circulating in the media associated the coronavirus disease with the market, where some animals, described through media images, were being sold as human food.

The early association of COVID-19 with Chinese food contributed to the perceived negligible risk to far away communities in East Africa. In relation to this, initial memes, video clips and messages that circulated through social media insinuated doubts about modes of COVID-19 infection, as there were alternative hypotheses linking the new coronavirus SARS-CoV-2 outbreak in China to transmission from animals to human beings (3–6). Specific connection of the transmission to the consumption of bats and snakes in China exacerbated misgivings in the discourse about the coronavirus risk and susceptibility in Africa where the indigenous menus rarely included these animals. Similarly, intensified coronavirus infection with fatality that seemed not clearly accounted for, particularly in Italy, Spain and Iran, from December 2019 to mid-March 2020, further shaped the perceived low risk of the pandemic in Africa. Conflicting views persist regarding differential coronavirus risk patterns in relation to predisposing demographic characteristics and underlying health conditions relative to the African population structure. The fact that the incidence of COVID-19 was diagnosed more among adult males of a particular age category elsewhere (7–10) dominated the discourse on differential risk and susceptibility patterns that would be lower in Africa. Recent popular discourses on the pandemic express a common public perception that Africa might be safer from extreme COVID-19 risk and fatality than other continents. However, precaution is often emphasised with regard to the possibility that the definition of demographic and underlying health conditions, especially comorbidities and gender factors (7,8) considered in health promotion may relegate the significance of potentially less vulnerable groups in the escalation of infection. Similarly, the perceived late arrival of COVID-19 and significantly lower testing rates in Africa (11) account for delays in preparedness for effective and culturally appropriate health promotion in East Africa.

Media images of frantic and violent reaction to the confirmed COVID-19 risk in Africa indicate the eventual political visibility of the pandemic threat (12). Similar coercive government approaches to enforcement of health promotion characterised interventions in the 2014 West African Ebola virus epidemic crisis and other infectious diseases elsewhere in Africa (13,14). Upon confirmation of the first COVID-19 cases in East and Southern Africa, the media reported many instances of people escaping from the rather violent government implementation of infection control protocols. Current social, cultural and political matrices thus influence mobilisation of the masses in health promotion initiatives during global pandemics such as COVID-19. This article draws on content analysis of musical and poetic compositions on coronavirus commonly shared among WhatsApp users in East Africa, to depict consequences of local sociocultural contexts for health promotion in spite of the pandemic. It explores the local construction of the meaning of COVID-19 and its implications for effective health promotion. Spontaneous musical and poetic compositions by both experienced and amateur artists are characterised as local attempts to enhance popular public participation in health promotion. The article hypothesises that the coronavirus lyrics that circulated through popular social media in East Africa since the confirmation of the first COVID-19 cases in East Africa, incorporate broad aspects of the cultural logic of care, which contextualises technical medical health promotion messages. The surge of artistic creation and the sharing of COVID-19 lyrics in social media, embody cultural adaptation to the pandemic crisis.

## Methods and materials

This article draws on a collection of short one to five minute coronavirus digital lyric videos shared through individual and group WhatsApp telephone contacts in Kenya, between March and mid-July 2020. These included compositions by local, regional and intercontinental artists, frequently shared on both WhatsApp and other social media channels. Followers’ comments about the lyrics, songs and associated artistic performances were recorded and transcribed. The local lyrics were mainly by amateur and professional composers from Kenya, Uganda, Tanzania and one each by Congolese and Burundian artists based in Kenya. A few of the catchy lyrics that circulated at the advent of coronavirus in East Africa were from Europe, the USA and South Africa. The video performances from Kenya are in English, Kiswahili, Luhyia and Giriama languages. Tanzanian lyrics are in Kiswahili language, while those by Ugandan artists are in English. The lyrics by East African artists involved occasional code-switching of English, Kiswahili, Luhyia and Luganda languages. A negligible number of audio-visual lyrics from Ghana and Nigeria were not included in the study as they were not shared more than once through the individual and group WhatsApp contacts that were available.

Forty-three digital video files of the lyrics and songs were identified and transcribed. The videos which had been posted more than three times by different WhatsApp users as either status updates or ordinary sharing were included in the collection. The selected lyrics were circulating concurrently on other social media channels, particularly YouTube, Facebook and Twitter. The location of some of the social media followers in East Africa were apparent in their YouTube and Facebook comments and general profile information on Facebook. Circulation of lyrics by celebrities was guaranteed by their numerous social media followers including fellow artists in East Africa and beyond. The social media postings of celebrity artists whose lyrics are included in this analysis had many views and likes on YouTube and Facebook, with hundreds of likes from followers across East Africa. Catchier amateur and celebrity lyrics were occasionally played on some television and radio stations in Kenya and widely circulated on social media when the coronavirus risk anxiety intensified in March to May 2020. The coronavirus digital lyrics that circulated in East Africa at the time reflect the urgency to produce songs for both health promotion and artists’ income as the disease catastrophically undermined their livelihood (15). Convenient samples of five contacts each in Uganda, Tanzania, Burundi and Rwanda confirmed the circulation of some of the lyrics included in the present analysis.

The lyrics were categorised by origin, language and genre and transcribed into English. Textual, content and discourse analyses were applied to determine the pattern of themes related to the objectives of the study. Emerging themes linked to the overall goal of the study were identified and have been presented in the present analysis and discussion. The basic premise is that folklore-stimulated artistic traditions, such as myths, narratives, proverbs, song and dance can affect health behaviors and work effectively toward promoting health education and wellbeing (16,17). The communicative power of folklore and folk art are embedded in specific idioms that are adaptable to the culturally relevant descriptions of health and illness-seeking behaviours. This was analysed with regard to how the artists flexibly switched codes in English, Kiswahili and local vernaculars in lyrical construction of coronavirus risks and creative demonstration of recommended COVID-19 control protocols. The folk-art improvisation for effective communication in this sense includes production of culturally appropriate idioms and symbols of distress, resilience and hope inspiration. This would be handy in relation to the uncertainty engendered by the perceived capricious coronavirus infection. As such folk art and related improvisations elaborate or dispel myths about threats to human wellbeing and symbolically present the realities of risk, prevention and disease management practices. Lyrical performances in the present analysis are therefore construed as socially generated improvisations for construction and interpretation of prevailing health emergency and associated livelihood realities. Combination of folk art, indigenous and modern lyrical genres in disease control communication provide contextualised symbolism for assigning meaning to health promotion messages.

## Results

### Lyric origins, composition languages and genres

Table 1 shows the origin and languages of the lyrical videos analysed.

**Table 1.** Lyric origins and performance language.

Composition origin	Performance language				Total
	English	English and other	Kiswahili	Ethnic vernacular	
Kenya	4	1	17	4	**26**
Uganda	2	2			**4**
Tanzania			4		**4**
South Africa	2	2			**4**
Europe	2				**2**
USA	2				**2**
Asia	1				**1**
**Total**	**13**	**5**	**21**	**4**	**43**

### Coronavirus lyrical genre improvisation

The shared digital videos represent either local African or adaptation of popular modern transnational genres. They include poetic narrations in English and Kiswahili languages, rap and reggae beats, rhumba, ‘bongo flava’, pop music, choir pieces and satire or humour lyrics. Initial and subsequent lyrics blend contemporary transnational with local genres or styles and poignant footage of the COVID-19 medical facts and emergencies. Some lyrics depict artistic ingenuity that has given rise to distinctive genres as spontaneous response to the perceived freak virus risk. It is conjectured that lyrics by artists from outside East Africa were regularly shared because they resonated with local fears, aspirations, copying needs and concerns about COVID-19 risks and public vulnerability. The adaptation by a South African artist’s *Toto-Africa* lyrics, dubbed *Toto-Africa (COVID-19 version)*, for instance, was widely shared via WhatsApp and Facebook in Kenya. WhatsApp users may have found the message partly quoted below appealing:

A lonely oke goes jollying Friday night …

He knows that he must do what’s right

But he ignores Cyril’s call for us to chill in quarantine

I don’t think you realise

COVID-19 is coming after you …

It doesn’t take a lot to be infected my bru …

Gotta take time to chill or it’s going to get real bad …

Reworked lyrics by a Hong Kong singer Kathy Makattack, entitled *Torn Parody* were also featured in the collection shared through WhatsApp in Kenya. It enacts the fright that was instilled locally by some of the COVID-19 prevention protocols:

I’m freaking out all right … I sterilize, I sanitize …

My hands are always frickin’ dry …

So then I finally went out on the street …

After days of being at home and hiding in my sheets

But then I start to worry ’cause …

… there’s nothing left at the grocery store

How do I use the lift?

How do I get to the door?

I hold on to my bags, cause all the germs fall to the floor.

Some of the lyrics reflect the attempt by non-East African musicians to neutralise excessive ‘COVID-19 fear’. Richard Williams (Prince EA), an American rap artist, for instance aims at easing the ‘panic response’ (18), which he calls the ‘fear virus’. His lyrics, which were shared by some WhatsApp users in Kenya, are partly quoted below:

… don’t be afraid …

There is good news during this tragedy …

To fight loneliness, people are performing concerts on their balconies

The UAE sent aid to Iran!

Japan donated supplies to China …

… we can’t let this DESTROY us …

All we ever really had in this world … was EACH OTHER …

Be alert, not fearful … the F virus is a pandemic easily transmitted …

Decontaminate yourself through dance, laughter and meditation …

… because TOGETHER is how we will rise above

… The only vaccine for this F virus, and every other virus, is LOVE …

South Africa’s Ndlovu Youth Choir song that circulated in East Africa mixes fun, indigenous rhythms and local Zulu language expressions as an artistic approach to dispelling myths about COVID-19. The choir restates the coronavirus symptoms and the infection control protocols in a cheering manner. Conversely, lyrics by the Morning Star group from South Africa include news extracts of dreadful COVID-19 related emergencies. The performance attempts to balance humour with solemn health promotion messages (16) on the reality of the coronavirus pandemic.

The early local lyrics in East Africa depict scepticism, spontaneity and fright in public responses to the COVID-19 pandemic and available interventions. The emergent local meanings of the disease that would hinder its effective control were also denoted in the preliminary coronavirus lyrics. The improvised lyrics and artistic performances served to affirm the reality of the COVID-19 risk and enhance public acquiescence in spite of emerging resistance to government enforcement of control protocols. The lyrics also signify the improvised role of digital lyric creation and sharing in the promotion of public resilience and socio-psychological wellbeing despite the perceived capricious COVID-19 trends.

### Reaffirming the COVID-19 risk

The construction and deconstruction of COVID-19 as a far-off pandemic feature in most of the lyrics analysed. The local communities in East Africa did not readily perceive the coronavirus risk and susceptibility to the disease. The local lyrics have therefore tended to re-emphasise the ominous reality of the global pandemic. The artists expressed the urgent need to resolve the indifference to the coronavirus infection prevention protocols. They sought to resolve the apparent denial among some local communities of the scientific truth about the indiscriminate coronavirus infection. The Kiswahili words of Bilinge musica du Congo, a Congolese band based in Nairobi, for instance, echo this sentiment in part: ‘Corona, where did you come from? You have jumped up to Africa.’ The performance stresses the need to stay home with family, wash hands and keep social distance as advised by the Kenyan president and the ministry of health.

Pioneer masterpieces tend to reiterate the fact that COVID-19 infection is not selective on the basis of race (colour), ethnicity or any other discriminatory attributes. The majority of such lyrics by the East African artists include footage from respective presidents’ periodic public speeches emphasising that ‘COVID-19 is real’. The lyrics incorporate the stern warnings from the presidents and ministers of health. The presidential and ministerial extracts inserted in some of the songs reflect the intention to communicate the responsibility of the government in safeguarding public health. A summary of this is represented well in the short reggae performance (in English, Kiswahili and Luganda languages) by Ugandan artists Bobi Wine and Nubian Li, titled *Corona Virus Alert*! The emphasis is summarised in the excerpt below:

The bad news is that everyone is a potential victim …

… the good news is that everyone is a potential solution

Sensitise the masses to sanitise

Keep a social distance and quarantine …

The corona virus is sweeping over mankind …

Everybody must be alert!

It’s a global pandemic that we can never take for granted …

Everybody must take charge …

The corona virus should not be taken lightly …

Everybody must be alert!

Discipline and personal hygiene …

Keep a distance from everyone

… Serious fever is a symptom

Dry cough is a symptom … Even sneezing is a symptom

Itchy eyes … and flu is a symptom

If you get sick … please do not transmit the disease

Isolate yourself from the public …

See what is happening in Italy …

Prevention is better than cure.

Most of the earlier lyrics present the objective facts of COVID-19, its symptoms and preventive measures, while echoing the inevitable local livelihood vulnerability.

### Reconstructing and interpreting coronavirus meaning

Improvised religious perspectives are incorporated in the lyrics to mediate the perceived sense of coronavirus fright and hopelessness. This situates the call for resilience in coping with COVID-19 in a discourse on unpredictability and uncertainty about prevention of infection. The health promotion lyrics adopting local Christian and Muslim styles thus underpin the gravity of the pandemic and the need for faith-based alert. This implies the need for synergy of mental, physical and social action sustained by the spirituality of local communities. The Kiswahili song titled *Corona*, by Paulo Siria, a Tanzanian artist, captures this perspective:

Let us all pray to God for this calamity …

If it must pass

Let us be careful and protect ourselves so that it does not befall us …

… the disease kills and has no cure and some of us are poor …

May the almighty God be the cure …

Our children in school, our mothers in markets

In transport vehicles, at work, we protect ourselves …

A similar approach is resonated by pioneer coronavirus lyricists in Kenya. Danny P Mboka, whose short *Kolona* (‘Coronavirus’) video has been popular since March 2020, reinforces the religious theme as indicated below:

Coronavirus … may it be defeated, in Jesus’ name …

We are crying, because of Corona, it is a very dangerous virus …

Which doesn’t have a cure … God save us from Corona …

It began in China, it has killed many people

Now it is spreading to the whole world and it is dreaded …

It is riskier than Cancer … than HIV/AIDS … Corona is creepy

To our president of Kenya …

You don’t allow these Chinese entry into Kenya …

Kenyans let’s pray, God to protect us,

Christians, let’s pray Corona will be defeated

Muslims let’s pray to God to protect us

All religions let’s pray, Corona will be defeated

Corona does not know the rich, may God protect us,

Corona doesn’t know the poor, Corona will be defeated …

It scares even Europe … may God protect us!

It scares America …

Lyrical messages embedded in religious belief underpin psychological resilience, but they may believe the medical rationality of health promotion. A performance in Kiswahili from Tanzania, titled *Corona Inapita* (‘Corona is passing’) illustrates this assertion. It includes verbatim quotes from the Tanzanian president’s key public messages as summarised in the extract below:

There is God … Tanzanians let’s know there is God …

Ebola came and it passed by …

… God does not sleep, he doesn’t doze off … Even Corona will pass

God protects his own …

[President’s comments: ‘I know, in the only way of prayer and relying on Almighty God, this Corona pandemic will also pass …’] …

… Government rise up and fight, the Lord of heaven is with you …

If they pray and humble themselves,

God will have mercy on their country (land)

Corona will cease … There is God …

Ebola came and it passed …

The largely religious and fatalistic masses tend to anticipate effective health promotion action through their faith in God and government stewardship. Faith in God, prayer and government responsibility for public health are thus given prominence in the construction of the actual meaning of the COVID-19 pandemic. The composition of the Kenyan State House Choir titled *Tumalize Korona Kenya* (‘Let us eradicate corona in Kenya’) represents an emerging construction of two-tiered health promotion agency mediated by spiritual devotion. First the religious genre and style of the lyrics urge the public to co-operate with the government by adhering to the COVID-19 control protocols. Second, the lyrics reiterate the ‘wake-up call’ theme that links success or failure in health promotion in spite of the global coronavirus pandemic to collective public commitment to spirituality that underpins societal wellbeing.

The improvised lyrics emerge as voices of culture brokers attempting to moderate public ambivalence about prescribed health promotion protocols. The lyrics deconstruct myths and misunderstandings about COVID-19. The ‘corona songs’ merge local health metaphors with the standard global public health facts through artistic improvisation of the health promotion messages in unique ethnic lyrical styles (15).

### Acquiescence and resistance to health promotion mobilisation

Narratives of acquiescence and resistance to health promotion mobilisation as depicted in the lyrics suggest at least five issues. First, insufficient grasp of the extent of coronavirus risk and vulnerability negate efforts to mobilise public participation. Despite the reports confirming an alarming increase of COVID-19 cases, sections of the masses persist ‘in denial’ of the imminent risk. Secondly, doubts and misconstruction of information on coronavirus risk belie the call for observance of safety protocols. Most of the East African masses are away from the epicentres of the coronavirus pandemic. This accounts for the fact that they lag behind global and local metropolises in envisaging the catastrophic outcome of the disease. Local artists improvised audio-visual lyrics with emergency footage of frightening hospital scenarios in China, Italy and America to enforce public compliance with the COVID-19 control protocols. Other artists include local hospital, ambulance and bereavement scenes in attempts to counter public indifference and denial about the risk of coronavirus infection in spite of persistent and ubiquitous precautionary messages. The lyrics and artistic improvisations help to resolve the challenge in mobilising the masses to appreciate the severity of COVID-19 and the meaning of social distancing as a way to save lives (19).

Thirdly, some of the lyrics include images that depict a paternalistic approach to enforcement of COVID-19 control protocols by government agents. These have included footage of what the general public perceived as desecration of bodies of coronavirus casualties through hasty burial and disposal practices that belied local funerary rites. In addition, some lyrics impute government culpability for public vulnerability to the coronavirus attack and illness in the first instance. This relates to the tendency to blame the government for abdication of its health system steward responsibility that would have ensured adequate protection from the ‘importation of the coronavirus’. From the political perspective, therefore, the government enforcement of health promotion measures manifest fear and panic that would possibly arise less from the risk of COVID-19 infection and more from the growing reality of its fallout (18). This scenario accounts for attempts by some artists to mitigate public mistrust and resentment, that seem to aggravate the perceived undue restraint on their autonomy, and to take charge of their own safety as they ensure a livelihood security. Some lyrical improvisations, therefore, reflect spontaneous dynamism and the artists’ realisation of the urgent need for both health education and inspiration of hope, while seizing the moment to capture their fans’ attention to send other messages through catchy tunes (15). This includes subtle expression of disapproval of the governments’ use of excessive force to ensure adherence to the coronavirus prevention protocols. The perceived use of force in health promotion for COVID-19 control initially undermined collaboration between the government and the public in Kenya and Uganda. Fourthly, insufficient harmonisation of government directives and community agency may undermine collaboration in coronavirus pandemic control. The digital lyrics attempt to remedy this by adjusting the communication to socially and culturally relevant expressions. Finally, resistance and acquiescence to COVID-19 related health promotion guidelines reveal public reaction to the perceived government indifference to the livelihood and social security encumbrance by the coronavirus control protocols.

## Quest for integrated health promotion

The lyrics shared on the COVID-19 pandemic in East Africa highlight contextual issues that need adequate culture expertise for effective health education and promotion. This requires careful translation of the medical logic (20,21) of the present health promotion guidelines in ways that are consistent with the context-specific logic of care (18,22). The concepts of medical logic of interventions and cultural logic of care are used here as heuristic frameworks for problematising the global health promotion guidelines for COVID-19 pandemic control in local social, cultural and economic contexts. Health education and promotion strategies need to consider the matrix of local cultures drawing on African experiences of other pandemics, such as HIV/AIDS and Ebola (18). Health promotion messages need to empower the masses to make choices that can support their pursuit of holistic individual and societal wellbeing. This is because the general public has different social, religious, cultural and political dynamics that influence the uptake of standard health promotion interventions (13,17). Creation and sharing of digital videos of lyrics are thus part of therapeutic art making, which has the potential to promote physical, mental and social health and livelihood security in spite of the COVID-19 pandemic crisis. Viewing, making and sharing music, various artistic improvisations and digital videos can serve as therapeutic vehicles for empowerment, solidarity and collective action as the masses strive to adopt the fundamental health promotion practices to save lives from the spread of COVID-19 (19). Lyrical initiatives in East Africa reflect both the agency of artists in translating health promotion messages to the public and the creation of resources for psychosocial coping. This highlights the need to enhance healthcare systems to support sustainable and integrated health promotion in local health systems. Frameworks for protecting those whose immune and social support systems are compromised by inequality (18), are also necessary.
